# Use of NSAIDs via the Rectal Route for the Prevention of Pancreatitis after ERCP in All-Risk Patients: An Updated Meta-Analysis

**DOI:** 10.1155/2018/1027530

**Published:** 2018-02-08

**Authors:** Lei-Min Yu, Ke-Jia Zhao, Bin Lu

**Affiliations:** ^1^Department of Gastroenterology, The First Affiliated Hospital of Zhejiang Chinese Medical University, Hangzhou, Zhejiang, China; ^2^The First Clinical Medical College of Zhejiang Chinese Medical University, Hangzhou, Zhejiang, China

## Abstract

The aim of this study was to assess the efficacy of the rectal administration of nonsteroidal anti-inflammatory drugs (NSAIDs) in preventing post-ERCP pancreatitis (PEP). We searched database for randomized controlled trials (RCTs) comparing periprocedural rectal administration of NSAIDs with placebo for the prevention of PEP. The rectal administration of NSAIDs significantly decreased the incidence of PEP in the whole patient population (odds ratio (OR): 0.44, 95% confidence interval (CI): 0.30–0.64, *P* < 0.0001), high-risk patients (OR: 0.34, 95% CI: 0.19–0.58, *P* = 0.0001), and all-risk patients (OR: 0.51, 95% CI: 0.31–0.84, *P* = 0.008). The incidence of PEP was reduced by indomethacin (OR: 0.54, 95% CI: 0.36–0.82, *P* = 0.004) and diclofenac (OR: 0.27, 95% CI: 0.15–0.46, *P* < 0.00001). The administration of NSAIDs before (OR: 0.42, 95% CI: 0.25–0.73, *P* = 0.002) or after (OR: 0.39, 95% CI: 0.27–0.56, *P* < 0.00001) ERCP reduced PEP. The NSAIDs were associated with a reduction in mild PEP (OR: 0.55, 95% CI: 0.36–0.83, *P* = 0.004) and moderate-to-severe PEP (OR: 0.47, 95% CI: 0.28–0.79, *P* = 0.004). The rectal administration of NSAIDs reduced the incidence of PEP in high-risk and all-risk patients.

## 1. Introduction

Since the introduction of endoscopic sphincterotomy, endoscopic retrograde cholangiopancreatography (ERCP) has become an important tool for the treatment of biliary and pancreatic diseases. The most common complication of ERCP is acute pancreatitis. The incidence of post-ERCP pancreatitis (PEP) ranges from 1% to 10% in all-risk patients, and it can even reach 25% in high-risk patients [1]. Most cases of PEP are mild, but about 10% of the episodes may be severe, resulting in significant morbidity and occasional mortality [2].

Multiple institutions have tried to develop mechanical and pharmacological procedures for the prevention of PEP; however, in general, the results of pharmacological interventions have been disappointing [3]. The drug somatostatin has been demonstrated to be useful [4], but it requires continuous infusion and is inconvenient to use in routine clinical practice. So, its use is limited. Nonsteroidal anti-inflammatory drugs (NSAIDs) have been found to be beneficial in the prevention of PEP. They act by suppressing the production of several main classes of proinflammatory lipids (e.g., leukotrienes, prostaglandins, and platelet-activating factor), resulting in the inhibition of phospholipase A2 activity [5, 6]. In addition, neutrophil-endothelial cell attachment is also inhibited by NSAIDs [6].

In 2014, the European Society for Gastrointestinal Endoscopy recommended the routine use of 100 mg of indomethacin or diclofenac via the rectal route immediately before or after ERCP in all patients without contraindications (recommendation grade A) [7]. But currently, there are no American Endoscopy Society guidelines that specifically recommend the use of rectal administration of NSAIDs to prevent PEP in all patients. In previous randomized controlled trials (RCTs) and meta-analyses, the results supported the use of rectal administration of NSAIDs for the prevention of PEP. However, some recently published RCTs have shown conflicting results [6, 8]. We hypothesized that differences in the risk factors for PEP may have led to the conflicting results. In other words, the efficacy of rectal administration of NSAIDs in preventing PEP may be affected by different risk populations. In order to clarify the role of rectal administration of NSAIDs in both high-risk and all-risk populations, we conducted this meta-analysis including all RCTs reported to date.

## 2. Materials and Methods

This meta-analysis was registered at the International Prospective Register of Systematic Reviews (number CRD42016039540).

### 2.1. Search Strategy and Quality Assessment

We performed a literature search of PubMed, Embase, and the Cochrane Library to find potentially relevant studies published from inception to May 2016. We selected articles adopting a highly sensitive search strategy in order to identify reports of RCTs, with a combination of medical subject headings and text words that included the following: (i) cholangiopancreatography, endoscopic retrograde; (ii) pancreatitis; and (iii) anti-inflammatory agents, nonsteroidal or indomethacin or ibuprofen or diclofenac. We carried out recursive searches as well as cross-referencing, adopting a “similar articles” function. Studies using oral and/or intramuscular injection and/or intravenous NSAIDs were excluded. Nonrandomized trials and studies with any research on rescue therapy, insufficient data on clinical response, pediatric studies, and duplicate publications were also excluded. Regardless of the primary outcome, we considered all potentially eligible studies for assessment. Additionally, we performed a manual search using the reference lists of crucial articles published in English.

### 2.2. Trial Selection Criteria

Inclusion criteria for study selection were as follows: (i) the study design included human trials published in English as full paper articles with randomized, blinded, and controlled trials; (ii) the study population included adult patients undergoing ERCP; (iii) the intervention included rectal administration of NSAIDs; and (iv) the comparison intervention was placebo. We required that the patients in the trial should have been given rectal administration of NSAIDs or placebo at random, before, during, or immediately after ERCP.

Two independent investigators (Lei-Min Yu, Ke-Jia Zhao) reviewed study titles and abstracts. Studies that satisfied the inclusion criteria underwent full-text assessment. The two investigators (Lei-Min Yu and Ke-Jia Zhao) analyzed the selected trials and extracted data. A third investigator (Bin Lu) resolved any disagreements. We used the Cochrane Collaboration tool to assess the risk of bias and evaluated the methodological quality of the included studies [9].

### 2.3. Outcome Measures

The primary outcome was the incidence of PEP. The secondary outcomes included the incidence of mild PEP as well as moderate-to-severe PEP. Subgroup analyses were performed based on the type of patients (high-risk and all-risk patients, separately), type of drug used (diclofenac versus control, indomethacin versus control), and the timing of drug administration (before, during, or immediately after ERCP).

### 2.4. Definitions

All-risk patients are all unselected patients undergoing ERCP. Patients who met one or more of the following major criteria were categorized as high-risk patients [10, 11]: clinical suspicion of sphincter of Oddi dysfunction (SOD), past history of PEP, pancreatic sphincterotomy, precut sphincterotomy, more than eight cannulation attempts, pneumatic dilatation of an intact biliary sphincter, ampullectomy, or pancreatography.

Patients who met two or more of the following minor criteria were also included in the high-risk group [10]: female patients younger than 50 years old, those with a recurrent history of pancreatitis (≥2 episodes), those who have received an injection of contrast agent into the pancreatic duct three or more times with at least one injection up to the pancreas tail, those who have received an injection of contrast agent excessively into the pancreatic duct causing pancreatic acini opacification, or those who acquired a cytological specimen from the pancreatic duct using a brush.

### 2.5. Statistical Analysis

We assessed the prophylactic effect of NSAIDs on the incidence of PEP by considering it as a dichotomous variable. For direct comparisons, random effects were adopted rather than the fixed effects model in order to take the heterogeneity between multiple studies into account. *P* values <0.05 were considered significant. To calculate the significance as well as the extent of statistical heterogeneity, the *I*^2^ index was used. A value greater than 50% was considered indicative of heterogeneity. Odds ratios (ORs) as well as the corresponding 95% confidence intervals (CIs) were calculated for each analysis.

Funnel plots and the Egger regression asymmetry test were used to estimate the possibility of publication bias. Sensitivity analyses were performed to evaluate the robustness of the results by eliminating each individual study in turn from the whole and analyzing the remainder again on the basis of quality. Subgroup analyses were performed based on the type of NSAIDs (diclofenac versus control, indomethacin versus control), the risk of patients (high-risk and all-risk patients, separately), and the timing of drug administration (before, during, or immediately after ERCP).

All statistical analyses were performed with RevMan version 5.3 software, and publication bias was analyzed by STATA/SE 12.0 software.

## 3. Results

### 3.1. Characteristics of the Included Trials

**Figure 1:**
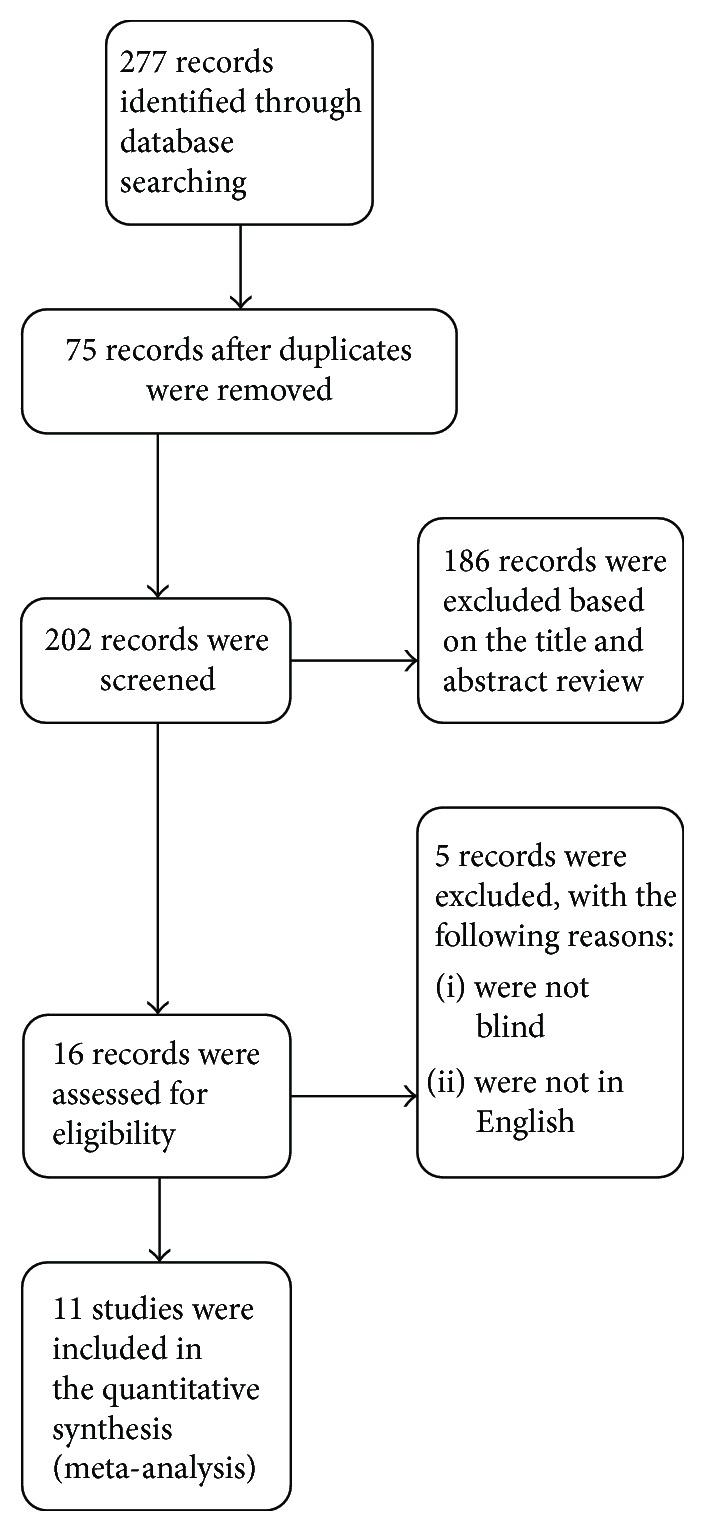
PRISMA flow diagram of included and excluded trials.

**Figure 2:**
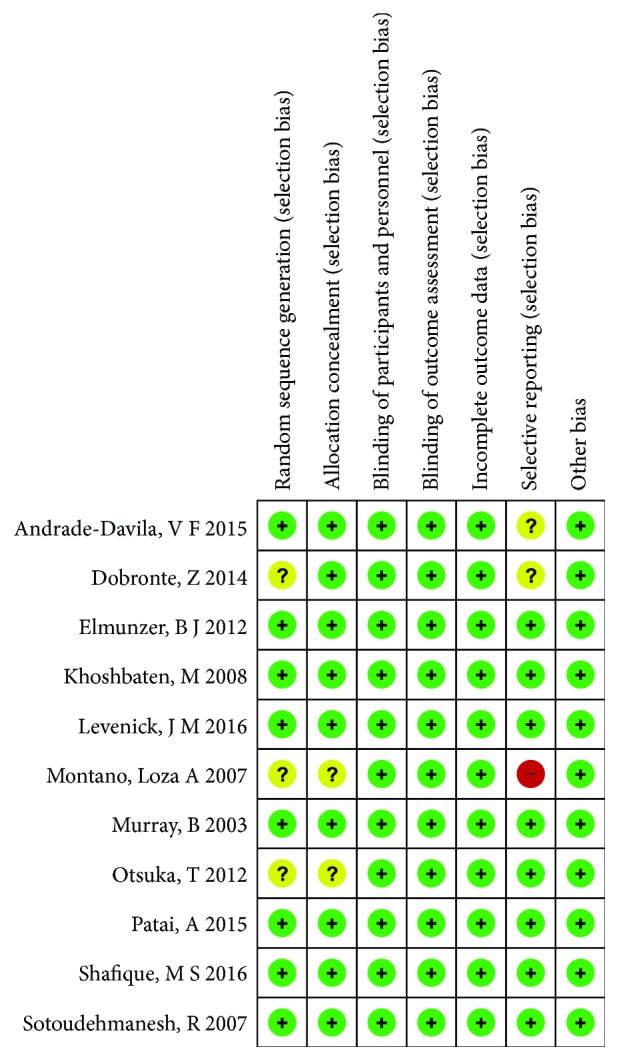
Consensus risk of bias assessments of the included studies. Green: low risk; yellow: unclear risk; red: high risk.

A total of 277 articles were identified, of which 75 duplicates were removed, 186 were excluded based on title and abstract review, 3 were removed for being nonblinded studies, and 2 were excluded for not being published in English (Figure 1). Finally, 11 RCTs met the inclusion criteria.  Table 1 shows the primary characteristics of the included studies in this meta-analysis, and Table 2 shows the outcome data of every included trial. The included studies were published between 2003 and 2016. The sizes of the RCTs varied from 100 to 665 patients, for a total of 3545 patients. PEP was defined by relatively similar criteria in all trials. Two different types of NSAIDs were used in the selected trials, including diclofenac [11–14] and indomethacin [6, 8, 10, 15–18].  Table 1 also shows that there were no reported adverse events attributed to NSAIDs in 10 trials. In one trial [15], itching in the anus in two patients in each group was found, and there was no mortality.  Figure 2 indicates the consensus risk from bias assessments of the included studies.

### 3.2. Publication Bias

**Figure 3:**
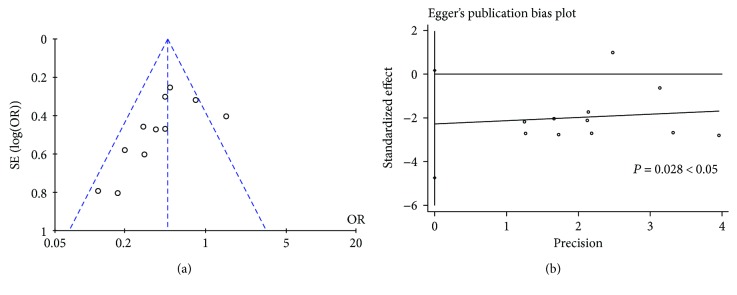
Bias assessment plot for the effect of rectal administration of NSAIDs in preventing PEP by a funnel plot (a) and Egger's test (b).

Visual inspection of the funnel plot test (Figure 3(a)) indicated that publication bias was possible. At the same time, Egger's weighted regression showed a mild publication bias for all analyses (*P* = 0.028, Figure 3(b)). In the comparisons, we were unable to assess publication bias because some negative results may not have been published.

### 3.3. Primary Outcome: The Incidence of PEP

**Figure 4:**
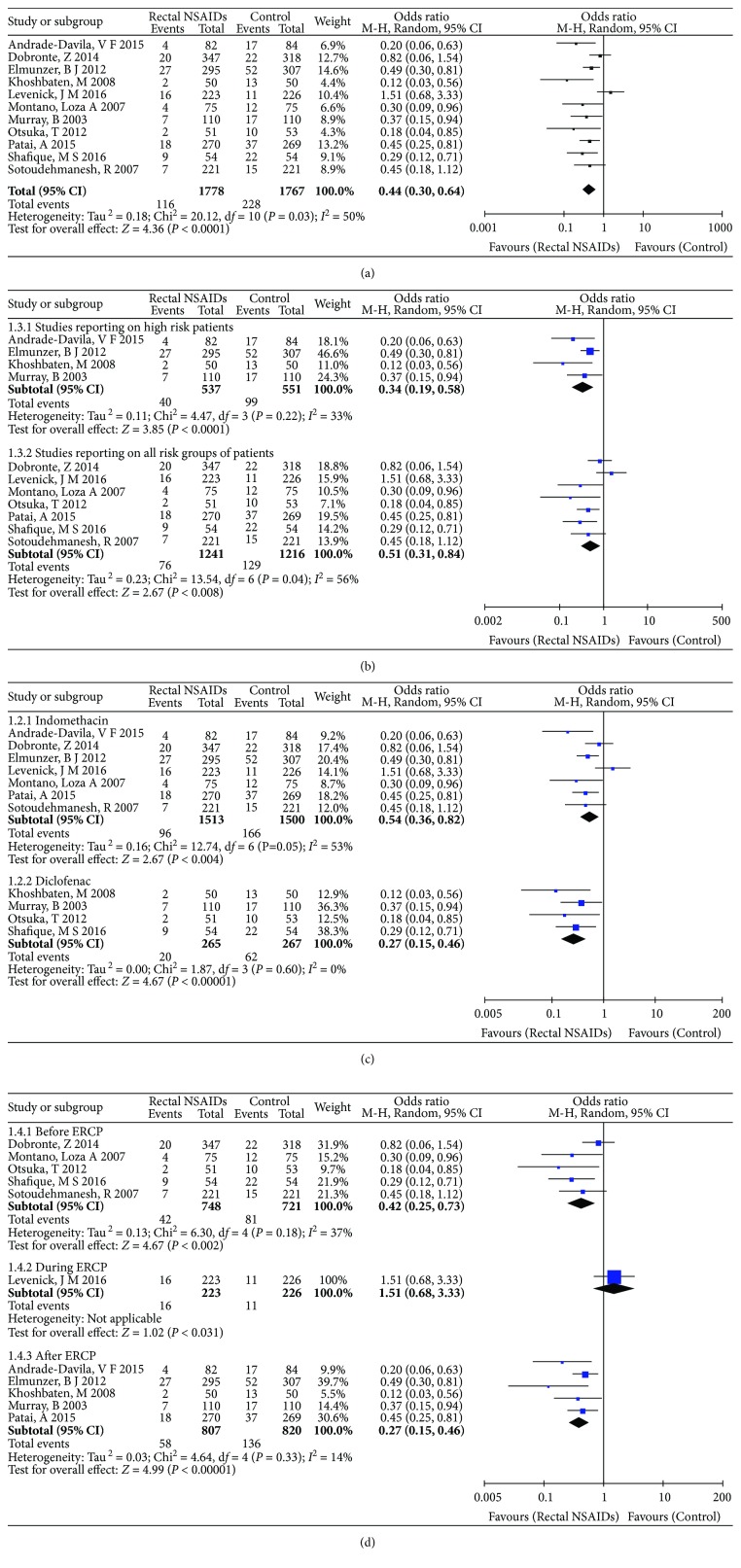
Primary outcome: (a) forest plot showing the effect of rectal administration of NSAIDs on the incidence of PEP; (b) subgroup analysis according to different risk patients; (c) subgroup analysis according to different drugs; (d) subgroup analysis according to the timing of drug administration.

Overall, 344 (9.70%) patients developed PEP: 116 in the NSAIDs group and 228 in the control group. The incidence of PEP was apparently reduced by the use of rectal administration of NSAIDs (OR: 0.44, 95% CI: 0.30–0.64, *P* < 0.0001; Figure 4(a)). Meanwhile, mild heterogeneity was detected in the analysis of the incidence of PEP (Tau^2^ = 0.18, Chi^2^ = 20.12, *P* = 0.03, *I*^2^ = 50%). Subsequently, sensitivity analysis was performed. After eliminating each study in turn, the results as well as heterogeneity were found to have disparity. More homogeneous results were achieved by excluding the outlier study of Levenick et al. [8], which was the source of heterogeneity (Tau^2^ = 0.05, Chi^2^ = 11.32, *P* = 0.25, *I*^2^ = 20%).

### 3.4. Subgroup Analyses

Subgroup analysis of studies reporting on high-risk patients and studies reporting on all-risk groups of patients was performed as shown in Figure 4(b). According to subgroup analysis of the four studies of high-risk patients [10–12, 15], the incidence of PEP was found to be statistically significant, favoring rectal administration of NSAIDs (OR: 0.34, 95% CI: 0.19–0.58, *P* = 0.0001). No heterogeneity in this subgroup was observed (Tau^2^ = 0.11, Chi^2^ = 4.47, *P* = 0.22, *I*^2^ = 33%). The incidence of PEP was also significantly reduced by NSAIDs for all-risk patients in the other seven studies (OR: 0.51, 95% CI: 0.31–0.84, *P* = 0.008) [6, 8, 13, 14, 16–18]. Mild heterogeneity was detected in the incidence of PEP in the all-risk patients group (Tau^2^ = 0.23, Chi^2^ = 13.54, *P* = 0.04, *I*^2^ = 56%).

Subgroup analysis of studies using indomethacin and diclofenac has been shown separately in Figure 4(c). Subgroup meta-analysis of seven studies [6, 8, 10, 15–18] showed that rectal administration of indomethacin was superior to placebo in preventing PEP (OR: 0.54, 95% CI: 0.36–0.82, *P* = 0.004). There was mild heterogeneity for the analysis of the incidence of PEP in the indomethacin subgroup (Tau^2^ = 0.16, Chi^2^ = 12.74, *P* = 0.05, *I*^2^ = 53%). Another subgroup meta-analysis of four studies [11–14] showed that rectal diclofenac was apparently superior to placebo in preventing PEP (OR: 0.27, 95% CI: 0.15–0.46, *P* < 0.00001), and there was no evidence of heterogeneity for these outcomes (Tau^2^ = 0.00, Chi^2^ = 1.87, *P* = 0.60, *I*^2^ = 0%).

Subgroup analysis of the timing of drug administration (before, during, or immediately after ERCP) is shown in Figure 4(d). In five studies, NSAIDs were administered before ERCP [6, 13, 14, 16, 18]. The meta-analysis of these studies showed that the incidence of PEP was significantly decreased with this treatment (OR: 0.42, 95% CI: 0.25–0.73, *P* = 0.002). No heterogeneity in this subgroup was observed (Tau^2^ = 0.13, Chi^2^ = 6.30, *P* = 0.18, *I*^2^ = 37%). Another meta-analysis of five studies in which NSAIDs were administered after ERCP [10–12, 15, 17] showed that the incidence of PEP was decreased, favoring the use of rectal administration of NSAIDs (OR: 0.39, 95% CI: 0.27–0.56, *P* < 0.00001). No heterogeneity in this subgroup was observed (Tau^2^ = 0.03, Chi^2^ = 4.64, *P* = 0.33, *I*^2^ = 14%). Only one study by Levenick et al. [8] used NSAIDs during ERCP, and the result showed that they could not prevent PEP (OR: 1.51, 95% CI: 0.68–3.33, *P* = 0.31). This study was the source of heterogeneity.

### 3.5. Secondary Outcome: The Incidence of Mild PEP or Moderate-to-Severe PEP

**Figure 5:**
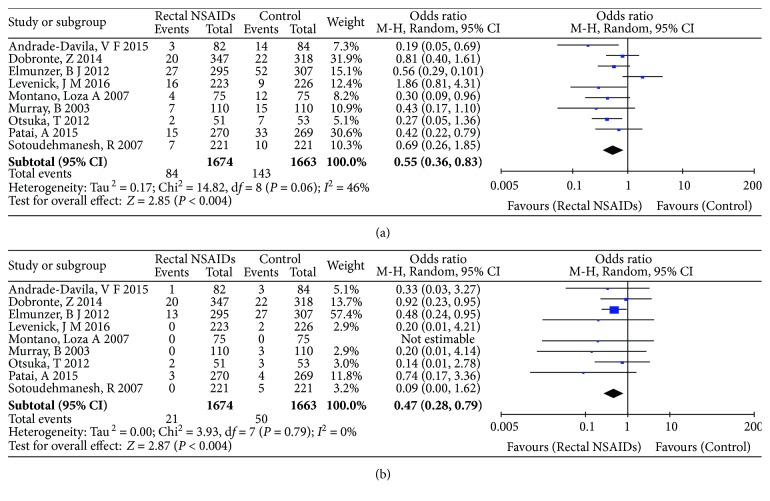
Secondary outcome: (a) forest plot showing the effect of rectal administration of NSAIDs on the incidence of mild PEP; (b) forest plot showing the effect of rectal administration of NSAIDs on the incidence of moderate-to-severe PEP.

As a secondary outcome, the incidence of mild PEP and that of moderate-to-severe PEP was analyzed separately (Figure 5). The incidence of different levels of severity of PEP was reported in nine studies [6, 8, 10, 12, 13, 15–18]. NSAIDs were associated with a decrease in the incidence of mild PEP (OR: 0.55, 95% CI: 0.36–0.83, *P* = 0.004) (Figure 5(a)). In these nine trials, there was no heterogeneity (Tau^2^ = 0.17, Chi^2^ = 14.82, *P* = 0.06, I^2^ = 46%). The incidence of moderate-to-severe PEP was also decreased with the use of NSAIDs (OR: 0.47, 95% CI: 0.28–0.79, *P* = 0.004), and there was no evidence of heterogeneity for these outcomes (Tau^2^ = 0.00, Chi^2^ = 3.93, *P* = 0.79, *I *^2^ = 0%) (Figure 5(b)). In other words, Figure 5 shows that rectal administration of NSAIDs reduces the incidence of mild PEP as well as moderate-to-severe PEP.

## 4. Discussion

Our results showed that, compared with placebo, rectal administration of NSAIDs as a prophylactic treatment can prevent PEP in high-risk and all-risk patients. Furthermore, based on the secondary outcome, NSAIDs can reduce the incidence of both mild and moderate PEP. Moreover, in all RCTs, there were no significant adverse events between the different groups, showing that a single dose of NSAIDs does not increase the danger of bleeding after ERCP (Table 1). The use of NSAIDs either before or after ERCP helps to reduce the incidence of PEP; however, more RCTs are required to determine the best timing of administration. Sensitivity analyses showed that the results remained stable when excluding the outlier study [8].

Complications of ERCP include pancreatitis (2.6%), bleeding (0.3%), infection (0.3%), cardiac (0.1%), pulmonary (0.1%), and bowel perforation (0.1%) [19]. Among these complications, PEP is regarded to be one of the main causes of mortality and morbidity. The onset is usually within 24 h of the procedure. Between the pancreatic injury during ERCP and the actual attack of symptoms is the “golden therapeutic window” (median time of 4.5 h), which creates a key treatment opportunity to prevent pancreatitis [20]. Due to the clinical and economic burden of PEP, extensive research efforts have been devoted to its prevention. The use of periprocedural rectal administration of NSAIDs is one of the most promising interventions.

To prevent PEP, pathogenic factors should be taken into consideration, such as the mechanical, thermal, hydrostatic, bacterial, and chemical insults accompanying cannulation and/or the injection of contrast medium into the pancreatic duct, or other modes of instrumentation of the papilla, as all the above may cause a pancreatic duct injury. All these pathogenic factors invoke an inflammatory response, which leads to development of pancreatitis. The intense inflammatory signaling mechanisms in acinar cells are important for the pathogenesis of pancreatitis [21]. Phospholipase A2 plays an important role by regulating some proinflammatory mediators, including arachidonic acid products and platelet-activating factors in the initial inflammatory cascade of acute pancreatitis [22]. It has been suggested that NSAIDs have beneficial effects on acute pancreatitis as they are potent antagonists of phospholipase A2 [23]. In addition, NSAIDs also inhibit nitric oxide synthase, which is involved in inflammation and cell damage [24]. The peak plasma concentration of NSAIDs is reached within 30 min after rectal administration [25], and bioavailability is complete. Based on the data available, it has been shown that only rectal administration of NSAIDs has an effect on preventing PEP, perhaps due to a more complete and rapid bioavailability than with the oral route [26, 27]. Gastric acidity can destroy drugs after oral administration [26]. Second, the metabolism of diclofenac is mediated by the cytochrome P450 (CYP) 2C9 enzyme (CYP2C9). Polymorphisms of the human CYP2C9 gene result in variable metabolic rates of diclofenac, thus influencing the efficacy of treatment [28].

In our study including 11 RCTs, 9 trials found that NSAIDs had a preventive effect on PEP, while 2 studies by Döbrönte et al. [6] and Levenick et al. [8] concluded that NSAIDs were not effective. Furthermore, the data provided by Levenick et al. [8] led to statistically significant heterogeneity to our analysis. Only Levenick et al. [8] used indomethacin during ERCP among these 11 studies. They found that the overall rate of PEP (6%) was consistent with their prestudy estimates, which is much lower than the mean rate of PEP in previous studies of high-risk patients [10]. This finding supports that the characteristics of the study population are of great importance and suggests that the conclusions drawn from prior studies are not generalizable to all patients undergoing ERCP. Recently, Luo et al. [29] conducted a RCT to compare routine use with selective application of transrectal indomethacin to prevent PEP. They concluded that the rectal administration of indomethacin before ERCP in all-risk patients was superior to giving the same drug only to high-risk patients after ERCP. Rectal administration of NSAIDs is clearly beneficial in the prevention of PEP in high-risk individuals; however, some studies found that NSAIDs were not effective in SOD patients. Senol et al. [30] noticed that diclofenac through the intramuscular route was only effective in preventing PEP in patients without SOD. These results were confirmed by Cheon et al. [26], who found that oral NSAIDs had no effect on the rate of PEP in more than half of SOD patients. Murray et al. [12] also found that rectally administered indomethacin was ineffective in SOD patients. This may be related to the fact that postmanipulation sphincter spasm or postsphincterotomy edema increases the pressure in the pancreatic duct, causing loss of the protective effects of NSAIDs in SOD patients. The intensity of the inflammatory cascade and the systemic response both determine the severity of pancreatitis. In addition, different risks of PEP in the study populations may influence the outcomes of RCTs. So, it is important to stratify patients based on their preprocedure and intraprocedure risk.

This study is an updated meta-analysis of 11 RCTs. Previously published meta-analyses have included only 7 RCTs [31–33]. Incidentally, in one of the meta-analyses [31], the dose of indomethacin used in Elmunzer's RCT was reported as 50 mg, despite the fact that two suppositories of 50 mg of indomethacin were used. This may have led to error in the reporting of the results.

In conclusion, we recommend the use of rectal administration of NSAIDs as cheap, globally available, easily administered, and safe medications for the prevention of PEP before or immediately after ERCP in all patients who are undergoing this procedure. A limitation of this meta-analysis was that non-English papers on this topic were not included, leading to a certain degree of selection bias. In addition, the funnel plot showed a mild publication bias in this meta-analysis. Therefore, there is a possibility that the results may be overestimated because our analysis included only published data. More studies are needed to evaluate the degree of the effect of NSAIDs on the prevention of PEP in patients with different risk factors.

## Figures and Tables

**Table 1:** Characteristics of studies included in the meta-analysis.

Study	Year	Country	Number of patients (NSAIDs/control)	Intervention	Definition of post-ERCP pancreatitis	Inclusion criteria	Study design	Adverse events attributed to rectal NSAID administration
Andrade-Dávila et al. [15]	2015	Mexico	166 (82/84)	Indomethacin (or placebo), rectally, 100 mg after ERCP	Amylase or lipase > 3 ULN within 24 h after ERCP and abdominal pain	High-risk patients	RCT TB MC	Itching in the anus in 2 patients in each group and there was no mortality
Döbrönte et al. [6]	2014	Hungary	665 (347/318)	Indomethacin (or placebo), rectally, 100 mg, before ERCP	Amylase and/or lipase > 3 ULN within 24 h after ERCP and abdominal pain	All-risk patients	RCT TB MC	None in both groups
Elmunzer et al. [10]	2012	USA	602 (295/307)	Indomethacin (or placebo), rectally, 100 mg after ERCP	Amylase > 3 ULN within 24 h after ERCP, new upper abdominal pain, and hospitalization for at least 2 nights	High-risk patients	RCT DB MC	None in both groups
Khoshbaten et al. [11]	2008	Iran	100 (50/50)	Diclofenac (or placebo), rectally, 100 mg after ERCP	Amylase > 4 ULN, epigastric or back pain, and epigastric rebound tenderness	High-risk patients	RCT DB SC	None in both groups
Levenick et al. [8]	2016	USA	449 (223/226)	Indomethacin (or placebo), rectally, 100 mg during ERCP	Lipase > 3 ULN within 24 h after ERCP and abdominal pain	All-risk patients	RCT DB SC	None in both groups
Montaño et al. [16]	2007	Mexico	150 (75/75)	Indomethacin (or placebo), rectally, 100 mg before ERCP	Amylase > 3 ULN, sharp pain radiating to the back, and nausea/vomiting	All-risk patients	RCT SB MC	None in both groups
Murray et al. [12]	2003	Scotland	220 (110/110)	Diclofenac (or placebo), rectally, 100 mg after ERCP	Amylase > 4 ULN, epigastric or back pain, and epigastric rebound tenderness	High-risk patients	RCT DB SC	None in both groups
Otsuka et al. [13]	2012	Japan	104 (51/53)	Diclofenac (or placebo), rectally, 50 or 25 mg, before ERCP	Amylase > 3 ULN within 24 h after ERCP and abdominal pain	All-risk patients	RCT DB MC	None in both groups
Patai et al.[17]	2015	Hungary	539 (270/269)	Indomethacin (or placebo), rectally, 100 mg, after ERCP	Amylase > 3 ULN within 24 h after ERCP	All-risk patients	RCT DB SC	None in both groups
Shafique et al. [14]	2016	Pakistan	108 (54/54)	Diclofenac (or placebo), rectally, 100 mg, before ERCP	Amylase > 4 ULN, epigastric pain with guarding and/or vomiting,	All-risk patients	RCT DB SC	None in both groups
Sotoudehmanesh et al. [18]	2007	Iran	442 (221/221)	Indomethacin (or placebo), rectally, 100 mg, before ERCP	Amylase > 3 ULN, epigastric or back pain, and epigastric tenderness	All-risk patients	RCT DB SC	None in both groups

NSAIDs: nonsteroidal anti-inflammatory drugs; ERCP: endoscopic retrograde cholangiopancreatography; ULN: upper limit of normal; SC: single center; MC: multicenter; SB: single blind; DB; double blind; TB: tripe blind; RCT: randomized controlled trial.

**Table 2:** Outcome data of studies included in the meta-analysis.

Study	Comparison	Number of patients	Severity of post-ERCP pancreatitis			Serum amylase (IU/L) 2 h after ERCP (mean ± SD)	Serum amylase (IU/L) 4 h after ERCP (mean ± SD)
			Incidence of post-ERCP pancreatitis	Mild	Moderate to severe		
Andrade-Dávila et al. [15]	Indomethacin	82	4/82	3	1	141.90 ± 92.60	NA
	Placebo	84	17/84	14	3	216.50 ± 105.20	NA
Döbrönte et al. [6]	Indomethacin	347	20/347	16	4	NA	NA
	Placebo	318	22/318	18	4	NA	NA
Elmunzer et al. [10]	Indomethacin	295	27/295	14	13	NA	NA
	Placebo	307	52/307	25	27	NA	NA
Khoshbaten et al. [11]	Diclofenac	50	2/50	NA	NA	310.28 ± 320.61	342.22 ± 331.65
	Placebo	50	13/50	NA	NA	667.80 ± 1034.15	948.86 ± 1296.69
Levenick et al. [8]	Indomethacin	223	16/223	16	0	NA	NA
	Placebo	226	11/226	9	2	NA	NA
Montaño et al. [16]	Indomethacin	75	4/75	4	0	148.22 ± 190.60	NA
	Placebo	75	12/75	12	0	240.73 ± 256.20	NA
Murray et al. [12]	Diclofenac	110	7/110	7	0	313.00 ± 398.54	321.00 ± 597.82
	Placebo	110	17/110	15	2	400.00 ± 702.70	507.00 ± 943.92
Otsuka et al. [13]	Diclofenac	51	2/51	2	0	NA	NA
	Placebo	53	10/53	7	3	NA	NA
Patai et al. [17]	Indomethacin	270	18/270	15	3	NA	NA
	Placebo	269	37/269	33	4	NA	NA
Shafique et al. [14]	Diclofenac	54	9/54	NA	NA	NA	NA
	Placebo	54	22/54	NA	NA	NA	NA
Sotoudehmanesh et al. [18]	Indomethacin	221	7/221	7	0	472.70 ± 910.40	NA
	Placebo	221	15/221	10	5	494.30 ± 694.10	NA

NSAIDs: nonsteroidal anti-inflammatory drugs; ERCP: endoscopic retrograde cholangiopancreatography; SD: standard deviation; NA: not available.
